# Long-term prognosis and risk factor-based surveillance strategy for patients with small gastric subepithelial lesion using endoscopic ultrasonography

**DOI:** 10.1097/eus.0000000000000166

**Published:** 2026-03-19

**Authors:** Jong Sun Park, Ji Hyun Park, Bumhee Park, Jong Hoon Park, Sun Gyo Lim, Sung Jae Shin, Kee Myung Lee, Sung Soo Ahn, Choong-Kyun Noh, Gil Ho Lee

**Affiliations:** 1 Department of Gastroenterology, Ajou University School of Medicine, Suwon, Republic of Korea; 2 Office of Biostatistics, Medical Research Collaborating Center, Ajou Research Institute for Innovative Medicine, Ajou University Medical Center, Suwon, Republic of Korea; 3 Solve-it Rheumatology and Gastroenterology Clinic, Yongin, Republic of Korea.

**Keywords:** gastric subepithelial lesion, GIST, lesion size, increasing rate, surveillance

## Abstract

**Background and Objectives:**

Initial surveillance strategy and subsequent monitoring for small gastric subepithelial lesions (SELs) lack a consensus. This study aimed to investigate the risk factors and performance of predictive markers for lesions requiring surgical intervention in patients with small gastric SELs.

**Methods:**

We retrospectively reviewed data of patients who underwent EUS at least twice between 2010 and 2023. Group S1 was defined as patients with a ≥20% increase in size from the initial EUS. The S1 was further divided into 2 groups according to requiring surgical intervention (S2 and S3).

**Results:**

Of the 476 patients, 106 (22.2%) had increase in size by ≥20% compared with the initial EUS (S1). The mean follow-up duration in the S1 was 70.2 months. Seven (1.5%) and 38 (8.0%) lesions were observed to have increased in size and were confirmed as gastrointestinal stromal tumors, respectively (S2). Lesions in the lesser curvature (odds ratio [OR]: 6.0, 95% confidence interval [CI]: 2.087–17.148, *P* < 0.001) and size of ≥12.8 mm (OR: 4.2, 95% CI: 2.083–8.542, *P* < 0.001) were risk factors for S2. Lesion size ≥12.8 mm is a key risk factor requiring timely intervention based on the Kaplan–Meier analysis. An increasing rate (0.7 mm/yr) showed superior performance as a predictive risk factor for S2 than having a lesion size ≥12.8 mm (area under the receiver-operating characteristic curve: 0.887 *vs.* 0.734, *P* < 0.001).

**Conclusions:**

Although the size of the SELs increased, the classification for group S2 was very important. These results emphasize the necessity for effective follow-up strategies, including timely interventions, for small gastric SELs.

## INTRODUCTION

Subepithelial lesions (SELs) are detected more easily owing to the widespread use of upper endoscopy for screening and surveillance.^[1,2]^ A multicenter study in Korea reported a prevalence of 1.6%, while another study found a prevalence of 1.9%.^[2–4]^ SELs are masses that usually protrude into the gastrointestinal (GI) lumen with an intact mucosal surface originating from the subepithelial layer.^[5]^ Most gastric SELs show benign features and are asymptomatic; however, some of these tumors display potentially malignant characteristics, such as GI stromal tumors (GIST) and neuroendocrine tumors.^[6,7]^

When endoscopists encounter a small SEL (<1 cm), they will not hesitate to perform annual upper endoscopy without the need for further evaluation and management; however, if gastric SELs have sizes of 1.1–2.0 cm, they need to decide whether to perform follow-up with additional EUS. To improve diagnostic accuracy, endoscopists may consider EUS-guided fine-needle aspiration biopsy for tissue acquisition when a nonlipomatous SEL is observed on EUS.^[2,7]^ There are many guidelines regarding the surveillance and management of gastric GIST ≤2 cm, but there is still a lack of consensus. The National Comprehensive Cancer Network and European Society of Medical Oncology guidelines recommend periodic endoscopic or radiographic surveillance for small gastric GIST (≤2 cm) if no high-risk features on EUS and endoscopic biopsy are observed.^[8,9]^ The American College of Gastroenterology clinical guidelines^[2]^ state that there is insufficient evidence to recommend surveillance *versus* resection for GIST ≤2 cm. According to the European Society of Gastrointestinal Endoscopy, lesions measuring 1–2 cm without a conclusive diagnosis require endoscopic surveillance at intervals of 1–2 years.^[10]^ In cases of confirmed GIST ≤2 cm, either ongoing monitoring or surgical removal is considered an acceptable approach.^[10]^

Although it is important to be able to screen SELs ≤2 cm, which increase in size or are highly likely to be GIST, early decision-making by endoscopists is very difficult in real-world practice. The optimal follow-up strategy for SELs that stop increasing in size remains unclear, leading to uncertainty in clinical decision-making. Current guidelines provide recommendations for SELs with continuous growth or high-risk features, but no consensus exists on the appropriate surveillance approach when growth stabilizes. To address this gap, we analyzed long-term follow-up data and categorized SEL growth into 2 distinct stages. This study aimed to identify risk factors and evaluate the performance of predictive markers for lesions requiring early surgical intervention.

## METHODS

### Patients

We retrospectively reviewed medical records of patients who underwent EUS exams at least twice for gastric SEL ≤2 cm at the Ajou University Hospital (Suwon, South Korea) between January 2010 and June 2023. The exclusion criteria were as follows: (1) tumor size >2 cm in the initial workup; (2) lesion originating from the third layer (submucosa layer) of the stomach wall; (3) undetermined origin; (4) lost to follow-up; (5) EUS examination only performed once within the specified period; and (6) SELs that underwent histologic sampling (EUS-guided fine-needle aspiration biopsy) during the initial EUS procedure.

### EUS workup

The endoscopic workup before EUS evaluation included the location, size, shape, surface characteristics, and response to forceps manipulation (rolling sign, cushion sign, tenting sign, and naked fat sign) of the lesion.^[11]^ EUS was performed using a 20-MHz, 2.5-mm-diameter catheter US probe (UM-3R; Olympus, Tokyo, Japan) and a radial ultrasound endoscope (GF-UM2000; Olympus), depending on the lesion size and accessibility. Especially, mini-probe EUS was used more for small lesions (<15 mm) to ensure higher resolution of superficial margins, while radial EUS was selected for larger lesions (≥15 mm) or those requiring deeper layer evaluation, including assessment of peri-gastric lymph nodes.^[12,13]^ The examination was performed using the water-filled balloon method after the lumen was filled with distilled water. The tumor size was measured at the maximal diameter using a caliper in the EUS image setting, with measurements taken along the longest axis of the lesion regardless of its shape. To ensure measurement consistency and minimize confounding from mucosal layer distortion, SEL size was reassessed using EUS at each follow-up visit. The homogeneity (homogeneous and heterogeneous) was defined as >75% of the total area, whereas the echogenicity (hyperechoic, isoechoic, and hypoechoic) was determined in comparison with a normal fourth layer (muscularis propria) of the stomach wall. The increasing rate was defined as the change in lesion size during the follow-up period divided by the total number of follow-up years. All EUS procedures were performed by expert endoscopists with at least 5 years of experience. To minimize interobserver variability, lesion size was measured using standardized caliper placement techniques at the maximal diameter regardless of lesion shape, and repeated measurements were performed in cases of uncertainty.

### Follow-up strategy and study design

All patients underwent follow-up EUS 6 months after the initial EUS and annually thereafter. However, in older patients or those with comorbidities, the decision for EUS was individualized to avoid unnecessary procedures. The lesions were classified into 2 categories: tumor size ≤1 cm and 1.1–2.0 cm. Increased size was defined as ≥20% increase in tumor diameter compared with baseline EUS measurement in accordance with standardized oncologic response criteria. This threshold was derived from the Response Evaluation Criteria in Solid Tumors (RECIST 1.1) guidelines,^[14]^ which define progressive disease as a ≥20% increase in the sum of target lesion diameters, with an absolute increase of at least 5 mm. Therefore, we defined group S1 as patients who experienced at least 1 episode of significant tumor growth (≥20% increase in diameter). In group S1, surgical removal was determined if the lesion size was >2 cm or accompanied by worrisome features on EUS. In addition, decisions for surgical resection were also made through multidisciplinary consultations involving gastroenterologists and gastrointestinal surgeons, particularly when the EUS findings were equivocal or when lesion behavior suggested malignant potential. Otherwise, the lesion was followed up with EUS. Among group S1, we defined group S2 as follows: (1) patients with GIST confirmed by pathology, or (2) lesions that were in group S1 but grew again after follow-up without removal. Patients with no further increase in size after observation in group S1 were defined as group S3. Therefore, group S2 had lesions that required surgical removal and was the target of our study, whereas group S3 had lesions that increased once but no longer increased afterwards.

### Histopathology workup

All SEL specimens obtained were subjected to a standard histopathological process, including hematoxylin and eosin staining, to evaluate tumor size, mitotic index, and ulceration. In our study, histopathologic diagnosis was based exclusively on surgically resected specimens. For the diagnosis of GIST, immunohistochemistry for c-KIT, CD34, and/or DOG-1 was performed.^[15]^ GIST were stratified as very low, low, intermediate, or high risk according to the National Institutes of Health consensus classification system.^[16]^

### Study outcomes

The primary outcome was the factors that differentiated the necessity of active intervention from observation. The secondary outcomes were the associated factors for each group, cumulative incidence for group S2 according to related factors, and the performance of predictive markers for group S2.

### Statistical analyses

The demographic characteristics of the patients were reported as mean (standard deviation) for continuous variables and as numbers and percentages for categorical variables. Characteristics were compared using an independent sample *t* test and the chi-square test or Fisher’s exact test for continuous and categorical variables, respectively. The receiver-operating characteristic (ROC) curve, which is a graphical representation of the true-positive rate *versus* the false-positive rate for all possible cutoff values, and the area under the curve were used to evaluate the diagnostic performance of lesion size as a risk factor for screening patients who require follow-up. The optimal cutoff value for lesion size was determined using the Youden index derived from the ROC curve analysis.^[17]^ This method identified the point that maximized the sum of sensitivity and specificity, thereby ensuring the best balance between the true-positive and false-positive rates. Univariate and multivariate logistic regression models were used to identify potential risk factors for screening patients requiring follow-up. The best models were determined using the forward, backward, and both-directions stepwise selection procedures for variable selection. The results were expressed as odds ratios (ORs) with 95% confidence intervals (CIs). Cumulative incidence curves were calculated using the Kaplan–Meier method and compared using the log-rank test. The incidence rates (per 100 person-years) and incidence rate ratios (IRRs) for group S2 were estimated across follow-up intervals using piecewise exponential models, including both time-fixed and time-varying formulations, to evaluate temporal patterns of event occurrence. Statistical significance was defined as *P* < 0.05. Statistical analyses were performed using R version 4.3.3 (R Project for Statistical Computing, Vienna, Austria).

## RESULTS

### Baseline characteristics

**Figure 1.**
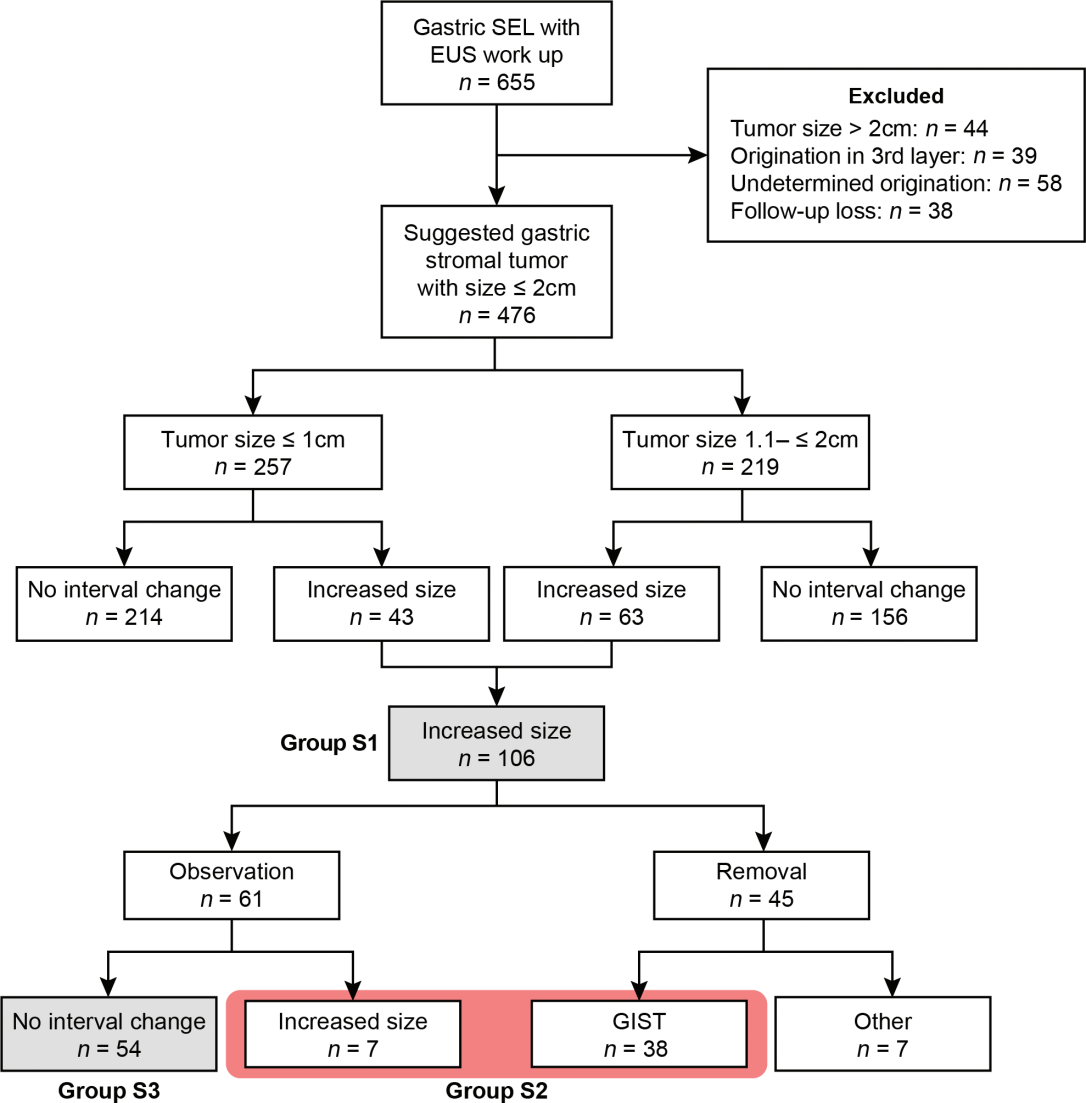
Flow chart of enrolled patients. GIST, gastrointestinal stromal tumor; SEL, subepithelial lesion.

Of the 476 included patients, 106 (22.2%) had lesions that increased by ≥20% compared with the initial EUS (group S1). Of the 106 patients, 45 (42.5%) underwent surgical removal, and 38 (35.8%) had confirmed GIST. Of the 61 (57.5%) patients in group S1 who did not undergo tumor removal, 7 (6.6%) showed an increase in size; thus, the 7 patients with an increase in size and the 38 patients with confirmed GIST were classified into group S2 (*n* = 45, 9.5%), with nonoperated 7 patients showing a mean lesion size 8.3 (2.6) mm. Of the total, 54 (11.3%) patients showed an increase in size compared with the initial workup, but there was no further change thereafter (Group S3) [Figure 1]. The mean age of all the patients was 63.8 (12.1) years and 308 (64.7%) were female. Most of the lesions were located in the upper third of the GI (*n* = 397, 83.4%), with a mean size of 10.3 (3.8) mm. Lesions >10 mm accounted for 45.8% (*n* = 218) of the total, and 14.1% (*n* = 67) of all lesions had worrisome features on EUS. Table 1 shows the overall baseline characteristics and comparison of the 2 groups categorized according to a significant increase in size compared with the initial EUS workup (group S1 *vs.* others). The proportion of inhomogeneous echogenicity was the only factor that was significantly higher in group S1 than in the other group (17.0% *vs.* 8.9%, *P* = 0.029). Although the mean lesion size did not differ between the groups, the proportion of lesions >10 mm in group S1 was 1.4-fold higher than in the other group (57.5% *vs.* 42.4%, *P* = 0.008). There was no significant difference in the prevalence of worrisome features between the 2 groups (15.1% *vs.* 13.8%, *P* = 0.854).

**Table 1:** Baseline characteristics of enrolled patients and comparisons between the 2 groups stratified according to changes in lesion size.

Variables	Total *N* = 476	No change *n* = 370	Change* (group S1) *n* = 106	*P* value
Age (yr), mean (SD)	63.8 (12.1)	63.7 (12.3)	64.0 (11.3)	0.848
Male sex, *n* (%)	168 (35.3)	126 (34.0)	42 (39.6)	0.346
Location 1 (longitudinal), *n* (%)				0.088
Upper 1/3	397 (83.4)	316 (85.4)	81 (76.4)	
Middle 1/3	43 (9.0)	29 (7.8)	14 (13.2)	
Lower 1/3	36 (7.6)	25 (6.8)	11 (10.4)	
Location 2 (cross-sectional), *n* (%)				0.094
Anterior	99 (20.8)	77 (20.8)	22 (20.8)	
Posterior	224 (47.1)	184 (49.6)	40 (37.7)	
Greater curvature	111 (23.3)	80 (21.6)	31 (29.3)	
Lesser curvature	42 (8.8)	29 (7.8)	13 (12.2)	
Multiple lesions, *n* (%)	99 (20.8)	76 (20.5)	23 (21.7)	0.902
Lesion size (mm), mean (SD)	10.3 (3.8)	10.1 (3.7)	10.9 (4.1)	0.054
Lesion size, *n* (%)				0.008
≤10	258 (54.2)	213 (57.6)	45 (42.5)	
10.1–20	218 (45.8)	157 (42.4)	61 (57.5)	
EUS finding				
Origination, *n* (%)				0.176
Second layer	13 (2.7)	8 (2.2)	5 (4.7)	
Fourth layer	463 (97.3)	362 (97.8)	101 (95.3)	
Echogenicity				0.137
Hypoechoic lesion	460 (96.6)	360 (97.3)	100 (94.3)	
Other	16 (3.4)	10 (2.7)	6 (5.7)	
Homogeneity				0.029
Homogeneous	425 (89.3)	337 (91.1)	88 (83.0)	
Inhomogeneous	51 (10.7)	33 (8.9)	18 (17.0)	
Presence of worrisome features, *n* (%)	67 (14.1)	51 (13.8)	16 (15.1)	0.854
Hyperechoic foci	65 (13.7)	51 (13.8)	14 (13.2)	
Irregular extraluminal border	2 (0.4)	1 (0.3)	1 (0.9)	
Cystic foci	1 (0.2)	0 (0.0)	1 (0.9)	
Adjacent malignant-appearing LN	0	0	0	

* Patients who experienced at least 1 episode of significant tumor growth (increase in diameter ≥20%).

LN, lymph node; SD, standard deviation.

### Risk factors for patients requiring intensive surveillance

Group S2 included the target lesion that was removed for histopathological confirmation. We performed logistic regression analysis to investigate the risk factors associated with group S2 [Table 2]. In the multivariate analysis, lesions in the lesser curvature (OR: 6.0, 95% CI: 2.087–17.148, *P* < 0.001) and lesion sizes ≥12.8 mm (OR: 4.2, 95% CI: 2.083–8.542, *P* < 0.001) were significantly associated with group S2. In contrast, lesion sizes ≥10.5 mm (OR: 1.7, 95% CI: 1.072–2.698, *P* = 0.024) and lesions in the middle 1/3 of the GI (OR: 2.1, 95% CI: 1.057–4.319, *P* = 0.034) were risk factors for group S1 in the multivariate logistic analysis (Supplementary Table 1, https://links.lww.com/ENUS/A391).

**Table 2:** Logistic regression analysis for determining risk factors for group S2.

Variables	Univariate		Multivariate	
	Odds ratio (95% CI)	*P* value	Odds ratio (95% CI)	*P* value
Age (yr)				
<50	Ref.		Ref.	
≥50	2.8 (0.648–11.736)	0.170	2.7 (0.613–12.287)	0.187
Female sex	0.7 (0.351–1.216)	0.179		
Location 1*				
Upper 1/3	Ref.			
Middle 1/3	2.7 (1.152–6.321)	0.022		
Lower 1/3	2.4 (0.913–6.106)	0.076		
Location 2†				
Posterior	Ref.		Ref.	
Anterior	3.7 (1.471–9.425)	0.006	2.5 (0.943–6.566)	0.066
Greater curvature	4.2 (1.731–10.285)	0.002	2.0 (0.768–5.290)	0.155
Lesser curvature	8.4 (3.101–22.960)	<0.001	6.0 (2.087–17.148)	<0.001
Multiple lesions	0.7 (0.293–1.570)	0.365		
Lesion size (mm)				
<12.8	Ref.		Ref.	
≥12.8	5.4 (2.831–10.231)	<0.001	4.2 (2.083–8.542)	<0.001
EUS echogenicity				
Hypoechoic lesion	Ref.		Ref.	
Other	4.8 (1.580–14.421)	0.006	2.8 (0.810–9.875)	0.103
EUS homogeneity				
Homogeneous	Ref.		Ref.	
Inhomogeneous	4.2 (2.034–8.680)	<0.001	2.1 (0.920–4.764)	0.078
EUS worrisome features‡	1.1 (0.486–2.668)	0.764		

* Longitudinal axis.

† Cross-sectional axis.

‡ Including hyperechoic foci, irregular extraluminal border, cystic foci, or adjacent malignant-appearing lymph node.

CI, confidence interval; Ref., reference.

### Long-term outcomes of group S2

**Figure 2.**
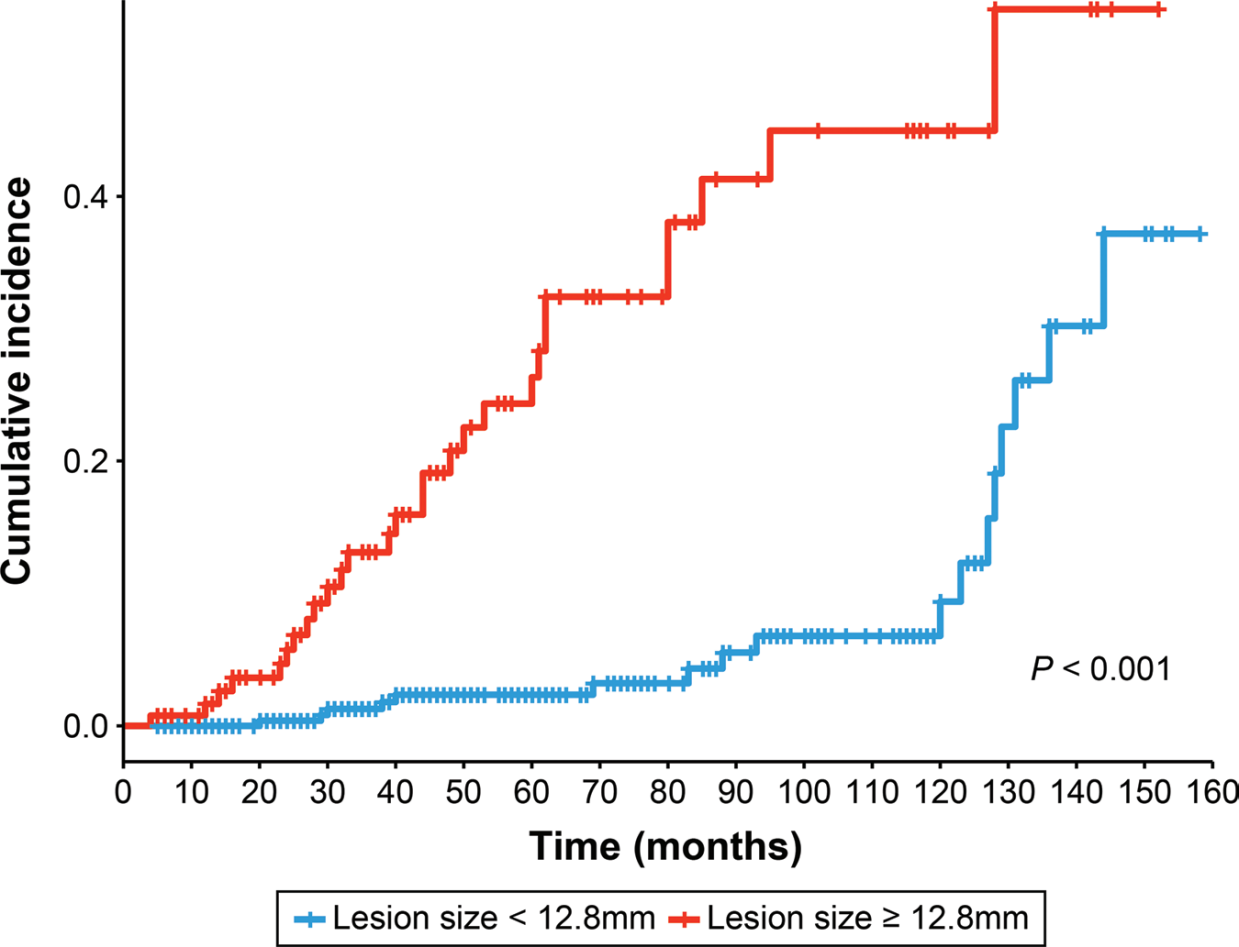
Kaplan–Meier analysis of cumulative incidence among patients with lesion size ≥ 12.8 mm *versus* those with smaller lesions.

The mean follow-up periods for groups S1 and S2 were 70.2 and 62.8 months, respectively. We used Kaplan–Meier curves to evaluate the cumulative incidence of events in group S2 based on 2 risk factors (tumor location in the lesser curvature and lesion size ≥12.8 mm). The location of the lesser curvature was not significant as the curves intersected (Supplementary Figure 1, https://links.lww.com/ENUS/A391); however, patients with lesion sizes ≥12.8 mm had a higher incidence rate than others (*P* < 0.001) [Figure 2]. Thus, patients with lesions ≥12.8 mm require more frequent and careful surveillance. To further quantify temporal changes in risk, the incidence rates (per 100 person-years) and IRRs for group S2 were estimated using piecewise exponential models (Supplementary Table 4, https://links.lww.com/ENUS/A391). The incidence rate progressively increased from 0.9 per 100 person-years during 0–2 years to 13.8 per 100 person-years beyond 10 years, indicating a rising long-term event occurrence. In the time-fixed model, the overall IRR beyond 10 years was 19.4 (95% CI: 7.013–53.573, *P* < 0.001) compared with the 0- to 2-year interval. The lesion effect for size ≥12.8 mm in the time-fixed model remained consistently stable across all intervals (IRR: 5.7, 95% CI: 3.125–10.471, *P* < 0.001), suggesting a persistent risk associated with larger lesions. In contrast, the time-varying model demonstrated dynamic risk patterns over time. The lesion effect was highest in the early period (IRR: 16.4 at 0–2 years, 11.0 at 3–5 years) and gradually diminished thereafter (IRR: 5.5 at 6–10 years, nonsignificant beyond 10 years). Meanwhile, the baseline interval effect markedly increased after 10 years (IRR: 88.1, 95% CI: 10.857–715.625, *P* < 0.001).

### Importance of distinguishing between groups S2 and S3

Groups S2 and S3 had increased lesion sizes compared with the initial EUS workup; however, group S3 no longer changed in size after the initial change (mean follow-up period: 81.4 months) [Table 3]. Of the total patients (*N* = 476), the highest proportion of lesions was located in the posterior (47.1%, *n* = 224) and lesser curvature (8.8%, *n* = 42); however, the posterior location was lower in group S2 than in group S3 (17.8% *vs.* 57.4%, *P* < 0.001). The mean lesion size in group S2 was 1.5-fold higher than in group S3 (13.2 ± 3.8 *vs.* 8.8 ± 3.4 mm, *P* < 0.001). The proportion of lesions >10 mm was also higher in group S2 than in group S3 (80.0% *vs.* 33.3%, *P* < 0.001). Inhomogeneous EUS findings were higher in group S2 than in group S3 (28.9% *vs.* 5.6%, *P* = 0.004).

**Table 3:** Comparison of baseline characteristics between groups S2 and S3.

Variables	Group S2 *n* = 45	Group S3 *n* = 54	*P* value
Age (yr), mean (SD)	63.3 (10.5)	65.9 (11.8)	0.258
Male sex, *n* (%)	20 (44.4)	19 (35.2)	0.464
Location 1 (longitudinal), *n* (%)			0.156
Upper 1/3	31 (68.9)	46 (85.2)	
Middle 1/3	8 (17.8)	5 (9.3)	
Lower 1/3	6 (13.3)	3 (5.5)	
Location 2 (cross-sectional), *n* (%)			<0.001
Anterior	12 (26.7)	9 (16.7)	
Posterior	8 (17.8)	31 (57.4)	
Greater curvature	15 (33.3)	12 (22.2)	
Lesser curvature	10 (22.2)	2 (3.70)	
Multiple lesions, *n* (%)	7 (15.6)	14 (25.9)	0.313
Lesion size (mm), mean (SD)	13.2 (3.8)	8.8 (3.4)	<0.001
Lesion size, *n* (%)			<0.001
≤10	9 (20.0)	36 (66.7)	
10.1–20	36 (80.0)	18 (33.3)	
Lesion size rate (mm/yr)	4.0 (5.8)	2.7 (3.0)	0.139
EUS finding			
Origination, *n* (%)			0.061
Second layer	0 (0.0)	5 (9.3)	
Fourth layer	45 (100.0)	49 (90.7)	
Echogenicity			0.089
Hypoechoic lesion	40 (88.9)	53 (98.1)	
Other	5 (11.1)	1 (1.9)	
Homogeneity			0.004
Homogeneous	32 (71.1)	51 (94.4)	
Inhomogeneous	13 (28.9)	3 (5.6)	
Presence of worrisome features, *n* (%)	7 (15.6)	9 (16.7)	1.000
Hyperechoic foci	5 (11.1)	9 (16.7)	
Irregular extraluminal border	1 (2.2)	0 (0.0)	
Cystic foci	1 (2.2)	0 (0.0)	
Adjacent malignant-appearing LN	0	0	

LN, lymph node; SD, standard deviation.

Additional logistic regression analysis was conducted to identify the factors associated with group S3 (Supplementary Table 2, https://links.lww.com/ENUS/A391). Lesion size <7.2 mm (OR: 3.5, 95% CI: 1.738–5.620, *P* < 0.001) and origin within the fourth layer of the GI (OR: 0.1, 95% CI: 0.042–0.455, *P* = 0.001) were associated factors for group S3.

### Lesion size and increasing rates as a predictor of group S2

**Figure 3.**
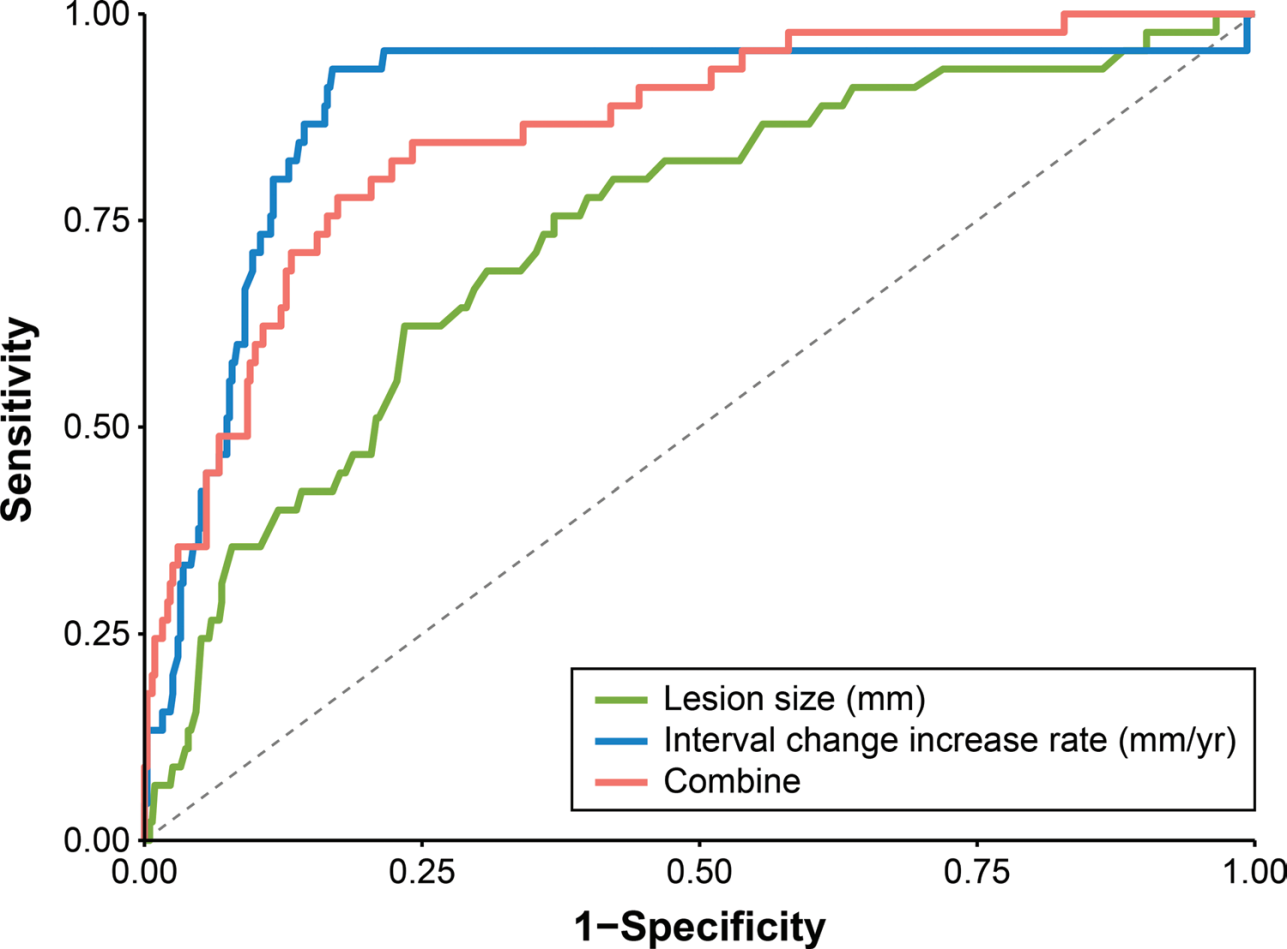
Receiver-operating characteristic curve of factors associated with a significant increase in subepithelial lesions requiring tumor removal. Lesion size (green line): the sensitivity, specificity, positive predictive value, and negative predictive value were 0.622, 0.766, 0.217, and 0.951, respectively. Increasing rates (blue line): 0.844, 0.861, 0.388, and 0.981, respectively. Combination (red line): 0.778, 0.826, 0.318, and 0.973, respectively.

Although a lesion size of ≥12.8 mm is important for identifying high-risk patients who require intensive surveillance (group S2), we compared its predictive value and combined it with increasing rates because lesion size increase can be transient. For lesion size alone, the area under the ROC curve (AUROC) for determining group S2 was 0.734 (95% CI: 0.666–0.812, *P* = 0.001) [Figure 3]. The cutoff value of 12.8 mm was identified as the optimal point based on the highest Youden Index (0.388), achieving the best balance between sensitivity (62.2%) and specificity (76.6%). In contrast, the AUROC of an increase rate of 0.7 mm/yr was 0.887 (95% CI: 0.827–948, *P* = 0.001). Even though a benefit was expected from combining lesion size and increasing rates, the AUROC value for this combination was only 0.857 (95% CI: 0.800–0.914, *P* = 0.001); therefore, if the lesion size is ≥12.8 mm, close surveillance is necessary, and if combined with an increasing rate of 0.7 mm/yr, preemptive removal should be considered.

### Comparison of Fletcher risk classification between group S2 and non-group S2 GIST

Among 43 patients with histologically confirmed GIST, group S2 (*n* = 38) lesions exhibited larger tumor sizes than those in non-group S2 (*n* = 5) (29.6 ± 10.6 *vs.* 18.6 ± 4.9 mm, *P* = 0.008, Supplementary Table 3, https://links.lww.com/ENUS/A391), and the proportion of tumors ≥2cm was higher in group S2 (86.8% *vs.* 40.0%, *P* = 0.039). In addition, the proportion of lesions classified as very low risk according to the Fletcher risk classification was significantly lower in group S2 than in non-group S2 (10.5% *vs.* 80.0%, *P* = 0.003), indicating that group S2 lesions of GIST were more likely to require surgical intervention due to higher malignant potential and aggressive behavior.

## DISCUSSION

Our study proposed a risk-stratified surveillance strategy wherein group S2 exhibited malignant potential or was histopathologically proven to be a GIST, necessitating immediate therapeutic intervention. By distinguishing between groups S2 and S3, we avoided unnecessary interventions and ensured a more targeted management of patients. Lesion size ≥12.8 mm and lesions at the lesser curvature were identified as significant risk factors for group S2, which required active surveillance or subsequent early intervention. For group S3, routine monitoring was appropriate for lesions originating in the second layer of the GI and measuring <7.2 mm in size. Recent studies have emphasized the importance of individualized management based on EUS morphology, lesion growth trends, or expert opinion without establishing clear thresholds.^[18,19]^ In real-world practice, because it is very difficult to make the next decision while tracking only the initial lesion size, we evaluated the rate of increase as a predictive marker of SEL requiring early intervention. However, our study provides specific cutoff values for lesion size ≥12.8 mm and growth rate ≥0.7 mm/yr that will guide surveillance and early intervention in small gastric SELs. Although a 0.7 mm/yr growth rate was strongly associated with malignant potential, its clinical significance may depend on lesion size. Nonetheless, the increasing rate was found to be superior to that of the lesion size; thus, even if the lesion size on initial EUS is <20 mm, if it is ≥12.8 mm and the rate of increase is 0.7 mm/yr at follow-up EUS, early intervention should be actively considered. Long-term follow-up studies of SEL, which is generally regarded as a benign disease, are currently lacking; thus, our results that were based on a long-term follow-up satisfy this gap in knowledge. Given that all patients were followed up using EUS, our results are highly reliable. Since surveillance and intervention strategies in this study were based on objective EUS criteria, symptomatic status was not a determining factor in patient management. In this study, intensive surveillance refers to continued EUS monitoring for high-risk lesions to ensure accurate size assessment and detect early tumor progression.

There are no established guidelines indicating the threshold lesion size at which GIST should be subjected to careful surveillance or timely intervention;^[2,8–10]^ hence, we proposed an evidence-based clinical guideline for different lesion size intervals. For lesion sizes <7.2 mm, patients warrant routine monitoring because they are 3.5 times more likely to belong to group S3. Lesions originating from the second layer of the GI showed a 10.0-fold higher possibility of being stable, which allows for a conservative approach. For lesion sizes of 7.2–10.5 mm, those that exhibited a growth rate exceeding 0.7 mm/yr are prone to developing into either increased growth or histopathologically confirmed GIST, which requires careful surveillance or subsequent intervention. The likelihood of requiring surgical or endoscopic intervention could increase if the lesion size exceeds 12.8 mm and is located on the lesser curvature; hence, these lesions should be closely monitored or treated with early intervention. Lesions in the 10.5–12.8 mm range have a higher likelihood of growth (group S1) but an uncertain risk of progression. Therefore, close monitoring with serial EUS is necessary to assess changes before determining the need for intervention.

There is no clear evidence-based clinical guidelines on critical periods requiring focused surveillance or the duration of follow-up for high-risk patients. This uncertainty promotes anxiety for both gastroenterologists and patients and increases the likelihood of loss to follow-up over time, potentially compromising patient outcomes. The Kaplan–Meier curves in our study demonstrated distinct periods of rapid event occurrence; hence, intensive surveillance during these clinically important intervals should be done to improve early detection, facilitate timely intervention, and ultimately reduce poor patient compliance.^[20]^ Unlike the initial rapid increase in events during the early follow-up period (within 3 years), significant lesion growth and malignant changes were observed at later intervals (60–70, 80–90, and 120–130 months). These findings suggest that surveillance should not be discontinued after 5 years to identify late-stage progression, and the clinical importance of timely management of high-risk lesions for long-term surveillance should not be underestimated.

Our study confirmed that tumor size was the most significant factor for progression; however, the key clinical question remains: should EUS surveillance continue indefinitely once the lesion exceeds 12.8 mm? The Kaplan–Meier curves, stratified by the 12.8-mm cutoff, revealed distinct temporal patterns. Larger lesions (≥12.8 mm) showed late-stage progression of peaks around 60–70 and 80–90 months, whereas smaller lesions (<12.8 mm) remained stable, with a plateau at a low cumulative incidence up to 120 months with a late increase thereafter. These Kaplan–Meier curves suggest that the early risk is mainly influenced by lesion size; however, time-dependent hazard persists in both groups over the long-term surveillance period. The analysis using a piecewise exponential model further clarified this pattern. For lesions ≥12.8 mm, the interval IRR (time effect) markedly increased in the late phase (beyond 10 years, 19.4 in the time-fixed model, 88.1 in the time-varying model, respectively), whereas the lesion IRR (size effect) remained significant only during the early intervals and then gradually diminished beyond 10 years. These findings in our study showed that early progression is lesion size-driven, whereas late progression arises from accumulated time-related changes or biological changes rather than the baseline tumor size itself. Therefore, EUS surveillance for lesions ≥12.8 mm should not be discontinued, and surveillance strategy in terms of follow-up duration and examination frequency should be deintensified due to a decreasing size effect, and individualized risk-stratified surveillance schedules are needed to capture late-stage progression driven by the time-dependent hazard. For lesions <12.8 mm, these smaller lesions served as the reference category in our model (lesion IRR = 1.0), and the interval IRR increased steadily across follow-up intervals both in the time-fixed and time-varying models beyond 10 years, showing that even initially small lesions have a time-dependent cumulative risk of being included in group S2 after long-term observation. Although most lesions <12.8 mm remain stable for years, this delayed, time-driven increase suggests that surveillance should not be stopped at 5 years. Instead, extending follow-up beyond 10 years at relatively longer intervals compared with the ≥12.8-mm group is recommended to capture the rare, late-occurring lesions that evolve into group S2 and require intervention while avoiding unnecessary over-surveillance. Although longer or more frequent follow-up in high-risk patients may have introduced surveillance bias, the consistent and pronounced late increase observed in both the Kaplan–Meier and IRR analyses suggests that these findings in our study cannot be attributed solely to differences in surveillance frequency or duration. Instead, it likely provides compelling evidence of a true biological, time-dependent accumulation of risk that persists even with long-term observation. In addition, the rate of size increase of 0.7 mm/yr as a predictive factor for group S2 showed better discriminatory performance than lesion size of AUROC 0.734 (95% CI: 0.666–0.812, *P* = 0.001) [Figure 3], suggesting the importance of incorporating growth dynamics when determining surveillance intensity and intervention decisions. We believe this study provides a solid starting point for larger, multicenter research to validate and generalize our observations and findings.

Our study had some limitations. First, this was a single-center retrospective study; however, we performed an analysis using various methods, covering >600 EUS data points. Second, higher dropout rates owing to the high cost and low accessibility of EUS equipment, predominantly used in tertiary hospitals, led to undesired challenges in the surveillance protocol. In a previous study, dropout rates of recommended surveillance reached up to 55.4%.^[21]^ Third, EUS measurements by different observers and probe selection (mini‑probe *vs.* radial probe) were subject to variability in measurement consistency. Although direct comparative studies focusing on size measurement variability are limited, probe-dependent discrepancies in layer delineation and subepithelial margin clarity have been reported and may indirectly affect maximal diameter measurement.^[12]^ Fourth, while we applied statistical methods to minimize overfitting, the relatively small number of events in group S2 may still pose a limitation. Further studies with larger cohorts are necessary to verify the model’s robustness in large populations. Fifth, multidetector computed tomography can serve as a complementary and adjunctive modality, particularly in situations where EUS is unavailable or impractical;^[22]^ however, EUS is recommended as the primary diagnostic tool for layer-specific analysis. Finally, fine-needle aspiration evaluation was not performed in all lesions. Further studies addressing these limitations should be performed.

## CONCLUSIONS

We attempted to distinguish between stable SEL lesions that showed a one-time change in size and significant SEL lesions that continued to increase in size and required surgical intervention. Tumor size ≥12.8 mm was the most important risk factor for surgical intervention. A growth rate of 0.7 mm/yr, along with lesion size, is helpful in deciding the necessity of early intervention. This study supports effective follow-up strategies, including timely intervention, in an era wherein gastric SEL is rapidly increasing owing to the expansion of screening endoscopy.

## Ethical Statements

The study protocol was approved by the Institutional Review Board of Ajou University Hospital (approval no. AJOUIRB-DB-2024-427). The requirement for written informed consent was waived owing to the retrospective nature of the study.

## Conflicts of Interest

The authors declare that they have no financial conflict of interest with regard to the content of this report.

## Author Contributions

Conceptualization: C.-K. Noh. Methodology: G. H. Lee and C.-K. Noh. Software: J. S. Park, J. Hoon Park, B. Park, and G. H. Lee. Validation: B. Park, G. H. Lee, and C.-K. Noh. Formal analysis: J. Hyun Park and B. Park. Investigation: J. S. Park, G. H. Lee, and C.-K. Noh. Resources: J. S. Park, G. H. Lee, and C.-K. Noh. Data curation: J. S. Park, J. Hyun Park, and B. Park. Writing—original draft preparation: J. S. Park, J. Hyun Park, and C.-K. Noh. Writing—review and editing: J. Hyun Park, S. J. Shin, and K. M. Lee. Visualization: J. S. Park, J. Hyun Park, and C.-K. Noh. Supervision: S. J. Shin, K. M. Lee, and S. S. Ahn. Project administration: G. H. Lee and C.-K. Noh. All authors have read and agreed to the final version of the manuscript.

## Data Availability Statement

The data that support the findings of this study are available from the corresponding author upon reasonable request.

## Supplementary Material


