# Supplementary material for: Long-term prognosis and risk factor-based surveillance strategy for patients with small gastric subepithelial lesion using endoscopic ultrasonography
Source: Endosc Ultrasound. 2026 Mar 19;15(1):62–70. doi: 10.1097/eus.0000000000000166 (PMC13048641; doi:10.1097/eus.0000000000000166)
Supplement: Supplementary file 1 [file eusj-15-62-s001.pdf]

Supplemental Digital Content

Title: Long-term prognosis and risk factor-based surveillance strategy for patients with small gastric subepithelial lesion using endoscopic ultrasonography

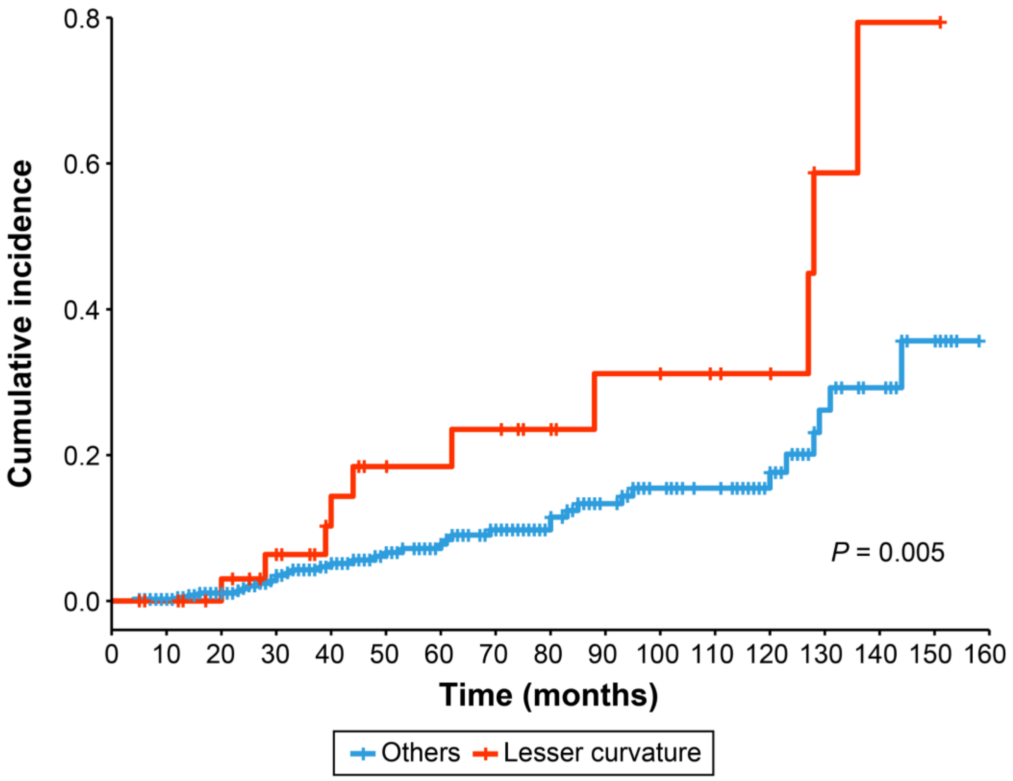

Supplementary Figure 1. Kaplan–Meier analysis of cumulative incidence among patients with lesions located on the lesser curvature vs. other locations. The two lines intersect and do not satisfy the proportional risk assumption; therefore, this finding is not statistically significant regardless of the *P* value.

Supplementary Table 1. Logistic regression analysis of risk factors in group S1

Variables	Univariate		Multivariate	
	Odds ratio (95% CI)	*P* value	Odds ratio (95% CI)	*P* value
Age, years				
<50	Ref.		Ref.	
≥50	1.6 (0.733–3.541)	0.236	2.0 (0.884–4.609)	0.095
Female sex	0.8 (0.504–1.228)	0.291		
Location 1[a]				
Upper 1/3	Ref.		Ref.	
Middle 1/3	1.9 (0.951–3.729)	0.069	2.1 (1.057–4.319)	0.034
Lower 1/3	1.7 (0.811–3.634)	0.158	1.3 (0.618–2.916)	0.456
Location 2[b]				
Posterior	Ref.			
Anterior	1.3 (0.733–2.357)	0.359		
Greater curvature	1.8 (1.041–3.051)	0.035		
Lesser curvature	2.1 (0.986–4.314)	0.055		
Multiple lesions	1.1 (0.633–1.814)	0.796		
Lesion size, mm				
<10.5	Ref.		Ref.	
≥10.5	1.9 (1.195–2.854)	0.006	1.7 (1.072–2.698)	0.024
EUS Origination				
2nd layer	Ref.			
4th layer	0.5 (0.143–1.394)	0.165	0.4 (0.118–1.216)	0.103
EUS echogenicity				
Hypoechoic lesion	Ref.			
Other	2.2 (0.766–6.087)	0.145		
EUS homogeneity				
Homogenous	Ref.			
Inhomogenous	2.1 (1.123–3.884)	0.02	1.8 (0.935–3.413)	0.079
EUS worrisome features[c]	1.1 (0.605–2.043)	0.732		

[a]Longitudinal axis.
[b]Cross-sectional axis.
[c]Including hyperechoic foci, irregular extraluminal border, cystic foci, or adjacent malignant-appearing lymph node.
EUS, endoscopic ultrasonography

Supplementary Table 2. Logistic regression analysis for determining factors associated with group S3

Variables	Univariate		Multivariate	
	Odds ratio (95% CI)	P value	Odds ratio (95% CI)	*P* value
Age, years				
<50	Ref.			
≥50	1.2 (0.455–3.162)	0.714		
Female sex	1.0 (0.556–1.819)	0.986		
Location 1[a]				
Upper 1/3	Ref.			
Middle 1/3	1.0 (0.376–2.680)	0.994		
Lower 1/3	0.7 (0.205–2.351)	0.557		
Location 2				
Posterior	Ref.			
Anterior	0.6 (0.284–1.362)	0.236		
Greater curvature	0.8 (0.371–1.533)	0.436		
Lesser curvature	0.3 (0.072–1.354)	0.120		
Multiple lesions	1.4 (0.722–2.667)	0.326		
Lesion size, mm				
<7.2	3.1 (1.738–5.620)	<0.001	3.5 (1.907–6.370)	<0.001
≥7.2	Ref.			
EUS Origination				
2nd layer	Ref.		Ref.	
4th layer	0.2 (0.060–0.602)	0.005	0.1 (0.042–0.455)	0.001
EUS echogenicity				
Hypoechoic lesion	Ref.			
Other	0.5 (0.066–3.954)	0.521		
EUS homogeneity				
Homogenous	Ref.			
Inhomogenous	0.5 (0.138–1.526)	0.204		
EUS worrisome features[a]	1.3 (0.583–2.704)	0.562		

[a]Longitudinal axis.
[b]Cross-sectional axis.
[c]Including hyperechoic foci, irregular extraluminal border, cystic foci, or adjacent malignant-appearing lymph node.
EUS, endoscopic ultrasonography

Supplementary Table 3. Comparison of risk classification of gastrointestinal stromal tumors between group S2 and non-group S2

	GIST in group S2 (n=38)	GIST in non-group S2 (n=5)	P value
Tumor size of GIST, mean (SD)	29.6 (10.6)	18.6 (4.9)	0.008
Tumor size (cm), n (%)			0.039
<2	5 (13.2)	3 (60.0)	
2.1–5.0	31 (81.6)	2 (40.0)	
5.1–10.0	2 (5.2)	0 (0.0)	
>10	0 (0.0)	0 (0.0)	
Mitotic index/50 HPF, n (%)			0.335
<5	26 (68.4)	5 (100.0)	
6–10	9 (23.7)	0 (0.0)	
>10	3 (7.9)	0 (0.0)	
Fletcher’s classification, n (%)			0.003
Very low	4 (10.5)	4 (80.0)	
Low	22 (57.9)	1 (20.0)	
Intermediate	7 (18.4)	0 (0.0)	
High risk	5 (13.2)	0 (0.0)	

GIST, GI stromal tumor; n, number; SD, standard deviation

Supplementary Table 4. Incidence rate and incidence rate ratio for metachronous recurrence according to follow-up interval

Interval (years)	No. events	Person-years (PY)	Incidence rate (per 100 PY)	IRR (95% CI)	*P* value	Lesion IRR (95% CI)	*P* value	IRR (95% CI)	*P* value	Lesion IRR (95% CI)	*P* value
				Time-fixed model				Time-varying model			
0–2	7	810.8	0.9	Reference	-	5.7 (3.125–10.471)	< 0.001	Reference	-	16.4 (1.980–136.306)	0.010
3–5	18	700.9	2.6	3.1 (1.313–7.529)	0.010	5.7 (3.125–10.471)	< 0.001	4.5 (0.500–39.949)	0.180	11.0 (3.614–33.359)	< 0.001
6–10	12	490.9	2.4	3.3 (1.284–8.307)	0.013	5.7 (3.125–10.471)	< 0.001	7.6 (0.888–64.952)	0.064	5.5 (1.737–17.244)	0.004
10+	8	57.8	13.8	19.4 (7.013–53.573)	< 0.001	5.7 (3.125–10.471)	< 0.001	88.1 (10.857–715.625)	<0.001	0.6 (0.078–5.134)	0.667

Abbreviations: PY, person-years; IRR, incidence rate ratio; CI, confidence interval

IRR = incidence rate ratio (exp [coef]), derived from the piecewise exponential model.

Lesion IRR refers to the IRR for lesion progression, estimated separately from the overall IRR.

Time-fixed model: covariates measured at baseline only

Time-varying model: covariates updated at each interval to reflect temporal changes

Reference category: the 0–2-year interval
